# Quality of life of Family Health Strategy professionals: a systematic review

**DOI:** 10.1590/1516-3180.2020.0661.R2.0902021

**Published:** 2021-05-26

**Authors:** Ana Carolina Chagas Pinatto Balabem, Murilo Navarro de Oliveira, Álex Moreira Herval, Ítalo de Macedo Bernardino, Walbert de Andrade Vieira, Renata Prata Cunha Bernardes Rodrigues, Luiz Renato Paranhos

**Affiliations:** 1 DDS. Master’s Student, Postgraduate Program on Public Health Management, Universidade Estadual de Campinas (UNICAMP), Campinas (SP), Brazil; II MSc. Doctoral Student, Postgraduate Program on Dentistry, School of Dentistry, Universidade Federal de Uberlândia (UFU), Uberlândia (MG), Brazil.; III PhD. Professor, Department of Preventive and Community Dentistry, School of Dentistry, Universidade Federal de Uberlândia (UFU), Uberlândia (MG), Brazil.; IV MSc. Doctoral Student, Department of Dentistry, Universidade Estadual da Paraíba (UEPB), Campina Grande (PB), Brazil.; V MSc. Doctoral Student, Department of Restorative Dentistry, Endodontics Division, School of Dentistry of Piracicaba, Universidade Estadual de Campinas (UNICAMP), Piracicaba, Brazil.; VI MSc. Professor, Department of Preventive and Community Dentistry, School of Dentistry, Universidade Federal de Uberlândia (UFU), Uberlândia, Brazil.; VII PhD. Professor, Department of Preventive and Community Dentistry, School of Dentistry, Universidade Federal de Uberlândia (UFU), Uberlândia, Brazil.

**Keywords:** Primary health care, Family health, Health Personnel, Basic attention, Primary healthcare, Health professionals, Health teams, Workers’ health

## Abstract

**BACKGROUND:**

Individuals’ quality of working life and motivation are directly related to their satisfaction and wellbeing. Although studies on the quality of life of family health workers have been conducted, there are none correlating these professionals’ wellbeing with this work model.

**OBJECTIVE:**

To review the scientific literature in order to identify the levels of quality of life, in their dimensions, of Family Health Strategy workers.

**DESIGN AND SETTING:**

Systematic review of observational studies developed through a partnership between two postgraduate schools (Piracicaba and Uberlândia).

**METHODS:**

The review followed the PRISMA recommendations and was registered in the PROSPERO database. Ten databases were used, including the “grey literature”. Two evaluators selected the eligible studies, collected the data and assessed the risk of biases, independently. The JBI tool was used to assess the risk of bias. A complementary statistical analysis was conducted on the means and standard deviations of the results from the WHOQOL-100 and WHOQOL-bref questionnaires.

**RESULTS:**

The initial search presented 1,744 results, from which eight were included in the qualitative analysis. The studies were published between 2007 and 2018. The total sample included 1,358 answered questionnaires. All the studies presented low risk of bias. The complementary analysis showed that the environmental factor (mean score 56.12 ± 2.33) had the most influence on the quality of life of community health workers, while physical health (mean score 14.29 ± 0.21) had the most influence on graduate professionals.

**CONCLUSION:**

Professionals working within the Family Health Strategy had dimensions of quality of life that varied according to their professional category.

## INTRODUCTION

Over the last decades, Brazil has faced the challenge of changing the public healthcare model, i.e. to migrate from the Flexnerian model focused on procedures and specialized care to a comprehensive care model based on understanding the social determinants of health.^1^^,^^2^ One of the crucial points in this change is to strengthen primary healthcare, for which the main operational strategy is the family health model.^3^^,^^4^^,^^5^ This strategy makes it possible to expand access to healthcare services and implement actions towards comprehensive healthcare.^6^^,^^7^

Expansion of this model has been associated with a 45% reduction in hospitalizations for conditions that are sensitive to resolution within primary healthcare, over a 15-year period.^5^ Data from the Ministry of Health indicated that in 2019 there were 43,754 family health teams operating throughout the country. These teams were responsible for providing primary healthcare to 64.47% of the Brazilian population.^8^

Family health work demands different skills for developing innovative community care practices, which makes the work complex and challenging.^9^ Primary healthcare professionals present high prevalence (52.9%) of chronic stress associated with their work.^10^ Analysis on this prevalence according to professional category shows that even higher prevalence can be observed: 54% among nurses and 67% among doctors.^11^^,^^12^ However, studies conducted among Brazilian professionals in family health teams have shown lower prevalence of burnout syndrome, varying according to the region of Brazil. In one municipality in the northeastern region, the prevalence of professionals with medium and high levels of burnout was observed to be 37.9%.^13^ In a municipality in southeastern Brazil, the prevalence of this syndrome reached 41.6%.^14^

In this regard, it is important to understand that the way in which work is organized affects both the workers’ quality of life and the service provided. These are therefore important objects of investigation.^15^^,^^16^ It can thus be seen that adequate provision of services requires maintenance of the quality of life of family health professionals.^17^

Individuals’ quality of working life and motivation are directly related to their satisfaction and wellbeing. Dissatisfaction in a team harms the evolution and productivity of the institution.^18^ Although studies on the quality of life of family health workers have been conducted, there are no studies correlating the wellbeing of these professionals with this work model,^19^ or proposing actions directed to the quality of life of these workers.

## OBJECTIVE

The aim of the present systematic review was to identify the levels of quality of life, in each of their dimensions, of Family Health Strategy workers.

## METHODS

### Protocol registration

This systematic review was performed in accordance with the Preferred Reporting Items for Systematic Reviews and Meta-Analyses (PRISMA)^20^ and the Joanna Briggs Institute Manual for Evidence Synthesis.^21^ The systematic review protocol was registered in the PROSPERO database under # CRD42019123243.

### Study design and eligibility criteria

This systematic review aimed to answer the following research question: “What are the levels of quality of life of professionals working in the Family Health Strategy?” This question was based on the “Population, Variable and Outcome” strategy, in which the population included in the study was primary healthcare professionals, the variable was the work in the Family Health Strategy and the outcome was quality of life, considering its different dimensions.

The inclusion criteria defined for selection of studies were that these should only be cross-sectional observational studies developed in Brazil, with quality-of-life questionnaires applied to professionals working in the Family Health Strategy. There were no restrictions on year or language. The following types of study were excluded: 1) experimental or non-cross-sectional studies; 2) studies that did not answer the research question; 3) studies on instrument validation; and 4) qualitative studies.

### Sources of information and search strategies

The primary study sources used were the PubMed (including MEDLINE), Scopus, Embase, SciELO, Web of Science, LILACS (Latin American and Caribbean Literature in Health Sciences) and Science Direct databases. The OpenThesis, OpenGrey, and OATD (Open Access Theses and Dissertations) databases were used to partially capture the “grey literature”. The MeSH (Medical Subject Headings), DeCS (Health Sciences Descriptors) and Emtree (Embase Subject Headings) resources were used to select adequate search descriptors. The Boolean operators “AND” and “OR” were used to enhance the research strategy through several combinations, as shown in Table 1. The search was performed in January 2020. The results obtained were exported to the EndNote Web™ software (Thomson Reuters, Toronto, Canada), in which duplicates were removed.

**Table 1.** Database search strategies

Database	Search strategy (January, 2020)
PubMed http://www.ncbi.nlm.nih.gov/pubmed	(“Quality of Life”[All Fields] OR “Health Related Quality Of Life”[All Fields] OR “Health-Related Quality Of Life”[All Fields] OR “Life Quality”[All Fields] OR “HRQOL”[All Fields]) AND (“Family Health”[All Fields] OR “Family Health Strategy”[All Fields] OR “Primary Health Care”[All Fields]) AND (“Health Occupation”[All Fields] OR “Health Worker”[All Fields] OR “Health Profession”[All Fields] OR “Health Personnel”[All Fields] OR “Occupational Health”[All Fields])
Scopus http://www.scopus.com/	(“Quality of Life” OR “Life Quality”) AND (“Family Health” OR “Family Health Strategy” OR “Primary Health Care”) AND (“Health Occupation” OR “Health Worker” OR “Health Profession” OR “Health Personnel” OR “Occupational Health”)
	(“Quality of Life” OR “Health Related Quality Of Life” OR “Health-Related Quality Of Life” OR “Life Quality” OR “HRQOL”) AND (“Family Health” OR “Family Health Strategy”) AND (“Health Worker” OR “Health Profession” OR “Health Personnel”)
LILACS http://lilacs.bvsalud.org/	(“Quality of Life”) AND (“Family Health”) AND (“Health Personnel”)
	(“Qualidade de Vida”) AND (“Saúde da Família”) AND (“Saúde do Trabalhador”)
	(“Quality of Life”) AND (“Family Health”) AND (“Health Workers”)
SciELO http://www.scielo.org/	(“Quality of Life”) AND (“Family Health”) AND (“Health Personnel”)
	(“Quality of Life”) AND (“Family Health”) AND (“Occupational Health”)
	(“quality of life”) AND (“Family Health”) AND (“health workers”)
Web of Science http://apps.webofknowledge.com/	((“Quality of Life” OR “Health Related Quality Of Life” OR “Health-Related Quality Of Life” OR “Life Quality” OR “HRQOL”) AND (“Family Health” OR “Family Health Strategy” OR “Primary Health Care”) AND (“Health Occupation” OR “Health Worker” OR “Health Profession” OR “Health Personnel” OR “Occupational Health”))
ScienceDirect https://www.sciencedirect.com/	(“Quality of Life” OR “Life Quality” OR “HRQOL”) AND (“Family Health” OR “Family Health Strategy” OR “Primary Health Care”) AND (“Health Occupation” OR “Health Worker” OR “Health Profession” OR “Health Personnel” OR “Occupational Health”)
Embase http://www.embase.com	(‘quality of life’ OR ‘health related quality of life’ OR ‘health-related quality of life’ OR ‘life quality’ OR ‘hrqol’) AND (‘family health’ OR ‘family health strategy’ OR ‘primary health care’) AND (‘health occupation’ OR ‘health worker’ OR ‘health profession’ OR ‘health personnel’ OR ‘occupational health’)
OpenGrey http://www.opengrey.eu/	(“Quality of Life”) AND (“Family Health” OR “Primary Health Care”) AND (“Health Worker” OR “Health Profession” OR “Health Personnel”)
	(“Quality of Life”) AND (“Family Health”) AND (“Occupational Health”)
OpenThesis http://www.openthesis.org/	(“Quality of Life” OR “Health Related Quality Of Life” OR “Health-Related Quality Of Life” OR “Life Quality” OR “HRQOL”) AND (“Family Health” OR “Family Health Strategy”) AND (“Health Worker” OR “Health Profession” OR “Health Personnel”)
OATD https://oatd.org/	(“Quality of Life” OR “Health Related Quality Of Life” OR “Health-Related Quality Of Life” OR “Life Quality” OR “HRQOL”) AND (“Family Health” OR “Family Health Strategy”) AND (“Health Worker” OR “Health Profession” OR “Health Personnel”)

### Study selection

The studies were selected in three stages. A calibration exercise was performed before the selection of studies, in which the reviewers discussed the eligibility criteria and applied them to a sample of 20% of the results retrieved to determine inter-examiner agreement. After an adequate level of agreement (kappa ≥ 0.81) had been reached, the first stage was started. In this, two reviewers (ACCPB and WAV) analyzed all the titles of the studies, independently. Any divergences between these examiners were discussed with a third reviewer (AMH) to reach a consensus. Studies that were not excluded in this phase continued to the next one. In the second phase, the same reviewers (ACCPB and WAV) read the abstracts, independently. The abstracts that did not meet the eligibility criteria were eliminated. Articles in which the titles met the objectives of the study but for which the abstract was unavailable were fully analyzed in the next phase. In the third phase, the preliminarily eligible studies were fully read to verify whether they met the eligibility criteria. In cases of disagreement between the two reviewers, a third one (AMH) was consulted to make a final decision. The studies rejected were registered separately, with explanations of the reasons for exclusion.

### Data collection

To ensure consistency between the reviewers in the data collection process, a calibration exercise was performed, in which the reviewers (ACCPB and AMH) extracted information from an eligible study together. After the selection, the studies were analyzed and the two reviewers (ACCPB and AMH) extracted the following information from each of them: study identification (author, year and location), sex, number of questionnaires answered, occupation, types of questionnaires used, mean results regarding quality of life obtained from the questionnaires, application of additional questionnaires and collection of socioeconomic data from the sample.

### Risk of individual bias of the studies

The risk of bias and individual quality of each study included were assessed using the JBI critical appraisal tools for use in systematic reviews on cross-sectional observational studies.^22^ Two authors (AMH and MNO) independently assessed each domain, in accordance with the PRISMA recommendations.^20^ The risk of bias was categorized as high when the study reached a “yes” score of up to 49%, moderate when the study reached a “yes” score of 50% to 69% and low when the study reached a “yes” score of more than 70%.

The question assessing the inclusion criteria for the study participants (Q1) was considered to have been answered “yes” (criteria verified) when the studies included the universe of family health professionals. The question referring to exposure factors (Q3) was considered “not applicable” because this systematic review aimed to identify factors that influence the quality of life, but only the dimensions most affected. Similarly, the questions about identification (Q5) and treatment (Q6) of the confounding factors were considered “not applicable” because they would identify the validity of the exposure studied.

### Qualitative synthesis and complementary statistical analysis

Data were extracted from the individual studies and then a synthesis of results was performed. Considering that all the eligible studies performed descriptive analyses to determine the levels of quality-of-life domains, without comparison between the groups, it was considered unviable to conduct a meta-analysis on continuous outcomes in order to estimate the effects of differences. Thus, the quality-of-life domains in the WHOQOL-bref questionnaire (physical, social, environmental and psychological) and WHOQOL-100 questionnaire (physical, psychological, level of independence, social, environmental and spiritual) were analyzed complementarily, considering the mean and standard deviation values expressed in the primary studies. It was possible to calculate means that were weighted according to the sample size of the scores reported in each study, with the aim of obtaining an overall estimate of the quality-of-life domains. Hence, the STATA software, version 15.0 (StataCorp, College Station, United States), was used.

## RESULTS

### Study selection

In the initial phase of study identification, after exploring the ten electronic databases, 1,744 results were found. Next, duplicate articles were excluded, which left 1,373 studies for the analysis on titles and abstracts. From these, 12 remained for full-text reading. After reading the full texts, a further four articles were excluded (Table 2).^17^^,^^23^^,^^24^^,^^25^

**Table 2.** Full texts excluded and reasons for exclusion

Author	Reason for exclusion
Martin et al.^25^	The instrument used in the study did not address quality of life
Fernandes et al.^17^	Instrument validation
Mota et al.^23^	Instrument validation
Ejlertsson et al.^24^	Duplicate publication

**Figure 1.**
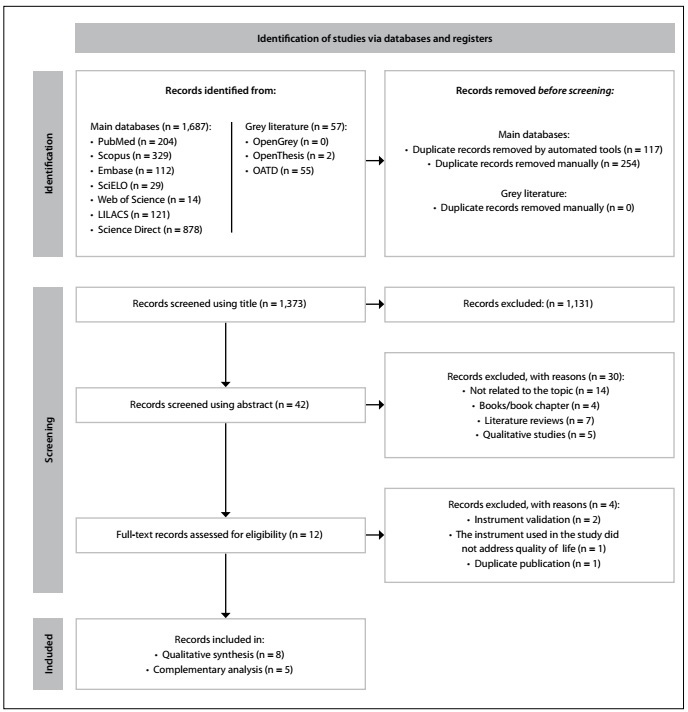
Flowchart of the literature search and selection process adapted from the PRISMA statement.

Thus, eight studies^26^^,^^27^^,^^28^^,^^29^^,^^30^^,^^31^^,^^32^^,^^33^ were selected for the qualitative analysis, but only five of these were retained for the complementary analysis stage. One of the three studies that were not retained for this final stage^27^ differed from the others regarding the instrument for measuring the quality of life. The other studies that were not retained^28^^,^^29^ did not present the data on quality of life in full. Figure 1 shows the entire process of identification, selection and eligibility of the studies.

### Characteristics of eligible studies

The eligible studies were published between 2007 and 2018. ^26^^,^^27^^,^^28^^,^^29^^,^^30^^,^^31^^,^^32^^,^^33^ The total sample included 1358 questionnaires answered by Family Health Strategy workers. Their average age ranged from 28 to 33 years (Table 3).^26^^,^^33^ All eight studies had been approved by ethics committees and the workers had signed an informed consent statement. The category of workers with the highest number of participants was community health workers (n = 557), but nurses (n = 180) and physicians (n = 162) also answered the questionnaires. All of the studies were conducted using questionnaires. Five studies^28^^,^^29^^,^^30^^,^^31^^,^^33^ used the WHOQOL-bref protocol, which is a reduced version of the WHOQOL-100 questionnaire, which was used in two studies.^26^^,^^32^ A single study used Walton’s Quality of Work Life scale (Walton’s QWL) as the methodology.^27^

**Table 3.** Summary of the main characteristics of the eligible studies

Author	State	Sample size (Total; %F; %M)	Number of questionnaires answered	Professionals assessed	Quality-of-life questionnaire	Other information collected
Kluthcovsky et al.^30^	Paraná	169; 89.3%; 10.6%	169	Community health workers	WHOQOL-bref	Not informed
Vasconcelos and Costa-Val.^31^	Minas Gerais	60; 96.7%; 3.3%	60	Community health workers	WHOQOL-bref	Socioeconomic data
Figueiredo et al.^27^	São Paulo	42; 100%; 0%	42	Community health workers	Walton’s QWL perception scale	Not informed
Miranzi et al.^32^	Minas Gerais	77; 54.8%; 45.2%	73	Physicians	WHOQOL-100	Additional questionnaire
Ursine et al.^33^	Paraná	77; 86.3%; 13.7%	73	Community health workers	WHOQOL-bref	Sociodemographic data
Fernandes et al.^26^	Minas Gerais	113; 92.2%; 7.8%	90	Nurses	WHOQOL-100	Sociodemographic data
Teles et al.^29^	Minas Gerais	797; 79.9%; 20.3%	762	Physicians, nurses, dentists, nursing technicians, oral health technicians and assistants, and community health workers	WHOQOL-bref	Sociodemographic and work data
Morais et al.^28^	Minas Gerais	122; 71.9%; 28.1%	89	Physicians	WHOQOL-bref	Sociodemographic work data and burnout questionnaire

F = female; M = male; WHOQOL = World Health Organization Quality of Life instrument; QWL = quality of work life.

### Risk of individual bias of the studies

All eight studies presented low risk of bias. The studies by Kluthcovsky et al.,^30^ Ursine et al.^33^ and Morais et al.^28^ obtained positive evaluations in all the criteria analyzed. The studies by Vasconcelos and Costa-Val,^31^ Figueiredo et al.,^27^ Miranzi et al.,^32^ Fernandes et al.^26^ and Teles et al.^29^ obtained positive evaluations for 80% of their questions. The question assessed as negative in these five studies^26^^,^^27^^,^^29^^,^^31^^,^^32^ related to the description of study location and subjects (Q2) because the studies did not inform these data, especially concerning study subjects (Table 4).

**Table 4.** Risk of bias assessed using the JBI critical assessment tool for systematic reviews, cross-sectional studies version^22^

Author	1	2	3	4	5	6	7	8	% Yes	Risk
Kluthcovsky et al.^30^	√	√	NA	√	NA	NA	√	√	100	Low
Vasconcelos and Costa-Val.^31^	√	--	NA	√	NA	NA	√	√	80	Low
Figueiredo et al.^27^	√	--	NA	√	NA	NA	√	√	80	Low
Miranzi et al.^32^	√	--	NA	√	NA	NA	√	√	80	Low
Ursine et al.^33^	√	√	NA	√	NA	NA	√	√	100	Low
Fernandes et al.^26^	√	--	NA	√	NA	NA	√	√	80	Low
Teles et al.^29^	√	--	NA	√	NA	NA	√	√	80	Low
Morais et al.^28^	√	√	NA	√	NA	NA	√	√	100	Low

1) Were the inclusion criteria in the sample clearly defined?; 2) Were the study subjects and scenario described in detail?; 3) Was exposure measured in a valid and reliable way?; 4) Were objective standard criteria used to measure the condition?; 5) Were confounding factors identified?; 6) Were the strategies to manage confounding factors informed?; 7) Were the results measured in a valid and reliable way?; 8) Was an adequate statistical analysis used?. √ = yes; -- = no; NA = not applicable.

### Result measurement and qualitative synthesis

The study by Figueiredo et al.^27^ used Walton’s QWL, which contains the following domains: adequate and fair compensation, working conditions, work capacities, work opportunity, social integration, respect for workplace laws, working life space and social relevance.^34^ These authors^27^ observed that the mean overall QWL score was 6.72 points, and fair compensation and working conditions were the domains most affected.

Table 5 ^26^^,^^30^^,^^31^^,^^32^^,^^33^ presents the results from extraction of the overall quality-of-life scores and the values obtained for each of the dimensions of the WHOQOL-bref and WHOQOL-100 questionnaires. Although these instruments were used in the studies by Teles et al.^29^ and Morais et al.,^28^ their data were not included in Table 5 because they were presented as percentages measured in the quality-of-life domains. The study by Teles et al.^29^ focused on assessing the results among professionals with low quality of life, and an overall score of 6.72 was obtained. These authors indicated that community health workers had moderate quality of life. Morais et al.^28^ observed that physicians presented unsatisfying quality of life in the physical, social and environmental domains and an overall score of 14.5 ± 2.2.

**Table 5.** Summary of the main results from the eligible studies included in the complementary analysis

Author	Overall quality of life	Dimensions assessed	Results	Main conclusions
Kluthcovsky et al.^30^	69.6 ± 14.5	Physical Social Environmental Psychological	74.2 ± 13.2 75.8 ± 14.2 54.1 ± 12.0 74 ± 11.4	The sociodemographic variables and the domains did not fully explain the variance in quality of life.
Vasconcelos and Costa-Val^31^	3.98 ± 0.65	Physical Social Psychological Environmental	82.8 ± 12 77 ± 18 76 ± 12.7 59.5 ± 12.5	The study presented negative results only for the environmental dimension.
Miranzi et al.^32^	Not informed	Physical Psychological Level of social dependence Environmental Spiritual	14.53 ± 2.35 15.32 ± 2.34 17.16 ± 1.95 15.67 ± 2.24 14.47 ± 1.76 16.67 ± 3.23	The worst results were found in the physical and environmental domains. The main complaints from the participants were lack of bonding, insecurity in the workplace, number of employment links and wages.
Ursine et al.^33^	76.7 ± 13.4	Physical Social Psychological Environmental	74 ± 12.3 71.5 ± 16.7 71.5 ± 13.6 58.0 ± 11.4	The environmental domain presented intermediate results, while the others showed positive results.
Fernandes et al.^26^	16.7 ± 2.2	Physical Psychological Level of social dependence Environmental Spiritual	14.1 ± 1.9 15.4 ± 2.0 17.0 ± 1.6 16.2 ± 2.1 14.2 ± 1.9 16.8 ± 2.6	The results of the questionnaire showed little or no negative impact on the domains.

### Complementary statistical analysis

Only five studies^26^^,^^30^^,^^31^^,^^32^^,^^33^ presented sufficient mean and standard deviation data for the complementary analysis. Three studies that were included in the descriptive synthesis^27^^,^^28^^,^^29^ were not included in this stage for the following reasons: one study used a different instrument,^27^ another study presented data on workers with low quality of life^29^ and another study described its data in a manner that prevented grouping in the complementary analysis.^28^

**Figure 2.**
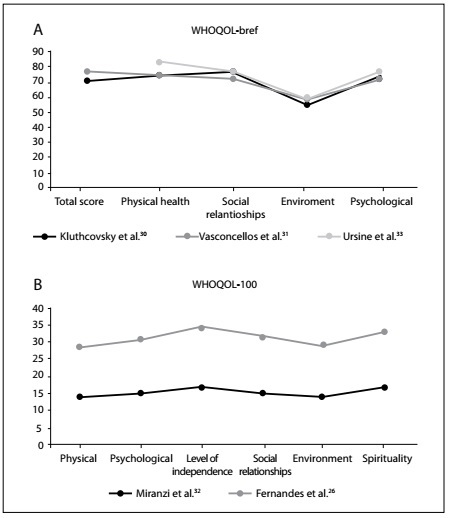
A) Levels of quality-of-life domains reported in the eligible studies based on the World Health Organization Quality of Life (WHOQOL)-bref questionnaire; B) Levels of quality-of-life domains reported in the eligible studies based on the WHOQOL-100 questionnaire.

Figure 2A shows the quality-of-life scores reported in the eligible studies based on the WHOQOL-bref questionnaire. Through estimating weighted means according to sample sizes, it was found that the total quality-of-life score from the WHOQOL-bref questionnaire was 71.74 (SD = 3.27). The environmental domain was the most affected (mean = 56.12; SD = 2.33), followed by the psychological (mean = 73.79; SD = 1.51), social relationships (mean = 75.00; SD = 2.03) and physical health domains (mean = 75.86; SD = 3.46).

Figure 2B presents the quality-of-life scores reported in the eligible studies based on the WHOQOL-100 questionnaire. Through estimating weighted means according to sample size, it was observed that the physical domain was the most affected (mean = 14.29; SD = 0.21), followed by the environmental (mean = 14.32; SD = 0.31), psychological (mean = 15.36; SD = 0.04), social relationship (mean = 15.92; SD = 0.31), spiritual (mean = 16.74; SD = 0.06) and level of independence domains (mean = 17.07; SD = 0.08).

## DISCUSSION

This systematic review aimed to identify the levels of quality of life among Family Health Strategy workers. The studies included mainly used the instruments developed by the World Health Organization (WHOQOL-100 and WHOQOL-bref). It was seen that graduate professionals and community health workers were affected differently regarding the quality-of-life domains. Most studies using WHOQOL-bref to investigate the quality of life of community health workers showed that the environmental dimension was the one most affected. However, the studies using WHOQOL-100 also included physicians and nurses, and indicated that the physical dimension was the one most affected.

The environmental dimension, which was most affected among community health workers, relates to freedom, safety, financial resources, access to health, social support, recreation, transportation and environmental quality.^35^ The qualitative studies by Souza and Freitas^36^ and Almeida, Peres and Fonseca^37^ corroborated this result, thus showing that community health workers felt unsafe when working with families because they were exposed to urban violence without any type of protection against this reality, which was present in several regions.

There was also a feeling of insecurity and uncertainty regarding the job, which was observed by Souza and Freitas^36^ and Figueiredo et al.,^27^ which helps to understand the low level of the environmental domain among community health workers. Another important factor in understanding the environmental dimension as the one most affected among community health workers is income (or availability of financial resources). The remuneration of community workers is the lowest among family health professionals^38^ and their monthly income may be considered to be close to^38^ or below^39^ the average wage reality of Brazilians, varying according to the region of the country. Therefore, considering that exposure to violence is an occupational risk for community health workers,^38^ these professionals should receive compensatory payment (hazard pay). Although this measurement does not have any direct impact on the quality of life, it may contribute to the remuneration for the work performed by this professional category. Another strategy for improvement of community health workers’ quality of life would be for their home visits to be made in pairs.

For physicians and nurses, the physical dimension presented the worst results. This dimension refers to pain, discomfort, sleep quality, fatigue, medication dependence and the ability to work.^35^ Physicians and nurses are the professionals working in primary healthcare among whom the highest number of studies on work burnout have been conducted.^40^ Compared with other primary healthcare professionals, they present the highest work stress.^42^ There is high prevalence of work burnout among graduate professionals.^40^^,^^42^ Silva et al.^42^ indicated that the prevalence of burnout was 64% and the prevalence of inability to work was 32% among nurses, physicians, dentists and social workers. Lima, Farah and Teixeira^40^ studied physicians, nurses and dentists working in the Family Health Strategy in a large city in the state of Minas Gerais, Brazil, and found that more than half of the professionals presented burnout syndrome.

The studies included in this systematic review used different instruments to assess the quality of working life, from unspecific ones (WHOQOL-100 and WHOQOL-bref) to a specific instrument for the work environment (Walton’s QWL). Regarding the unspecific instruments included in this systematic review, it is worth noting that both were developed by the same group of researchers: WHOQOL-bref is the short version of WHOQOL-100.^35^ The authors of these instruments suggested that both are effective in assessing quality of life within the concept determined by the World Health Organization, but that the short version would be indicated for assessing work routines in epidemiological studies.^35^

The existence of several instruments lies within the very essence of the concept of quality of life: polysemic, imperfect and dynamic.^43^ The specific instrument used by Figueiredo et al.^27^ (Walton’s QWL) comes from the conception of work-related quality of life that has been observed within a context of labor movements towards more legal certainty in the workplace, better working conditions and adequate remuneration.^34^ However, the creation of this concept, and consequently the instrument, was linked to a historical and cultural particularity of a region, with constant updates and new propositions for the concept of work-related quality of life.^44^ Therefore, the systematic review and meta-synthesis by Pennisi et al.^45^ indicated that assessing the quality of life of Family Health Strategy professionals should include the following factors: working conditions, work processes, interpersonal relationships, personal aspects, work context, work overload and autonomy.

This study is not free from limitations. The first of them related to the heterogeneity observed in the eligible studies, caused by the use of different questionnaires to assess the quality of life (WHOQOL-bref, WHOQOL-100 and Walton’s QWL), as previously discussed. Moreover, the results were presented differently (percentages or means and standard deviations), which prevented inclusion of a greater number of studies in the complementary analysis. Another limitation was that the studies were directed towards different professionals, who present professional and social particularities. Lastly, there was an important difference in the number of questionnaires answered in each study, ranging from 42 to 762, which may explain the heterogeneity in the findings. Thus, although the results obtained are consistent, they should be analyzed carefully and further studies are required, in order to assess the true impact of the working conditions of Family Health Strategy professionals on their quality of life.

## CONCLUSION

Quality-of-life domains are affected differently among primary healthcare professionals working in family health teams. While physicians and nurses are more affected in the physical domain, community health workers are affected in the environmental domain. This shows that actions in favor of the quality of life of family health professionals cannot be standardized, but the particularities of each professional category must be considered.

Another important factor is the influence of the region covered by the family health team on the quality of life of community health workers. There is an important paradox in considering this relationship and the promotion of quality of life for this professional category because the region is itself the workplace of community health workers, but is also the main factor responsible for interfering with their quality of life.
